# Effects of deep brain stimulation on personality and executive function in Parkinson’s disease

**DOI:** 10.1590/1980-5764-DN-2024-0254

**Published:** 2025-09-05

**Authors:** Thayná Laís de Souza Arten, Amer Cavalheiro Hamdan

**Affiliations:** 1 Universidade Federal do Paraná, Setor de Ciências Humanas, Programa de Pós-Graduação em Psicologia, Curitiba PR, Brazil.

**Keywords:** Parkinson Disease, Deep Brain Stimulation, Executive Function, Personality, Doença de Parkinson, Estimulação Encefálica Profunda, Função Executiva, Personalidade

## Abstract

Parkinson’s disease (PD) is associated with cognitive and personality changes that extend beyond its characteristic motor symptoms.

**Objective:**

To analyze the effects of subthalamic nucleus deep brain stimulation (STN-DBS) on executive function (EF) and personality (The Five-Factor Model — FFM) in PD patients, comparing outcomes between those treated solely with medication and those receiving both medication and STN-DBS.

**Methods:**

A total of 69 participants were divided into three groups: DBS, medication, and control. Evaluations included inventories, tests, and scales, with results summarized in tables highlighting sociodemographic variables and screening outcomes.

**Results:**

Cognitive assessments indicated that the DBS group exhibited slightly lower cognitive scores compared to the medication group. Personality differences were minimal, with only extraversion demonstrating significance.

**Conclusion:**

STN-DBS did not have a significant impact on executive functions or personality traits in patients with PD. These results highlight the importance of considering broader cognitive and neuropsychiatric factors when evaluating patient outcomes following DBS.

## RESUMO

A doença de Parkinson (DP) está associada a alterações cognitivas e de personalidade que vão além de seus sintomas motores.

**Objetivo:**

Analisar os efeitos da estimulação cerebral profunda do núcleo subtalâmico (STN-DBS) nas funções executivas (FE) e na personalidade (Modelo dos Cinco Fatores — FFM) em pacientes com DP, comparando os resultados entre os pacientes tratados exclusivamente com medicação em comparação àqueles tratados com medicação e STN-DBS.

**Métodos:**

Sessenta e nove participantes foram divididos em três grupos: DBS, medicação e controle. As avaliações incluíram inventários, testes e escalas, e os resultados foram resumidos em tabelas que destacam variáveis sociodemográficas e os resultados nos instrumentos.

**Resultados:**

As avaliações cognitivas mostraram que o grupo DBS apresentou pontuações ligeiramente inferiores em comparação com o grupo de medicação em relação à cognição. As diferenças de personalidade foram mínimas, com significância observada apenas para extroversão.

**Conclusão:**

O STN-DBS não apresentou impacto significativo nas funções executivas ou traços de personalidade em pacientes com DP. Esses resultados ressaltam a importância de se considerar fatores cognitivos e neuropsiquiátricos mais amplos na avaliação dos desfechos clínicos após a DBS.

## INTRODUCTION

 Parkinson’s disease (PD) is the second most common neurodegenerative disease worldwide^1^ , with 6.1 million individuals diagnosed with the disease in 2016^2 -5^. Pharmacological treatments for PD are primarily dopamine-based, but these sometimes fail to offer stable relief of motor symptoms or cause intolerable side effects^6^ . A therapeutic option for PD with levodopa-induced motor complications is implantation of deep brain stimulation (DBS) which reduces motor symptoms and can improve sleep and neuropsychiatric factors, such as impulsivity, anxiety, and depression, enhancing quality of life^7,8^. However, results are inconsistent, and some studies report changes in cognition, behavior, and personality^9,10^. 

 One theory used to describe personality is the Five Factor Model (FFM)^11-13^. PD patients have been described as introverted, apprehensive, tense, restless, and cautious, with higher levels of neuroticism and lower levels of both openness and extraversion^14,15^. These traits may be influenced by medication used in PD treatment, as levodopa and dopamine agonists are associated with impulsivity and apathy. Though personality traits are stable, drug effects and disease progression can alter them^5,16^. Some studies suggest DBS may affect cognition in certain cases, though these effects are typically rare and have minimal impact on quality of life^9^ . Recent studies have reported lower cognitive performance in patients treated with DBS, particularly affecting memory, verbal fluency, and executive functions (EF)^5,17,18^. 

 This study provides a comprehensive analysis of cognitive and personality changes in PD patients undergoing subthalamic nucleus (STN) DBS using the FFM as a framework for assessing personality traits. To the best of our knowledge, this is the first study to examine both domains concurrently. The aim of this study is to analyze the effects of STN-DBS on personality and EF in patients with PD. 

## METHODS

### Participants

 This study employed a cross-sectional design to analyze differences in EF and personality traits in 69 participants divided into three groups: 23 treated with both medication and DBS (DBS group; five with unilateral stimulation and 18 with bilateral stimulation in the STN), 23 treated with medication only (drug group), and 23 without PD (control). Given the nature of the study, no baseline evaluation was conducted, limiting the ability to assess changes attributable to DBS over time. The medications used in both PD groups were similar, including dopamine precursors, dopamine agonists, monoamine oxidase-B (MAO-B) inhibitors, NMDA receptor antagonists, anticholinergics, and catechol-O-methyltransferase (COMT) inhibitors. The total number of participants was predicted using the software G*Power^19^ considering an effect size of 0.40, significance level of 0.05, and sample power of 0.80. 

 The inclusion criteria for both PD groups were literate people aged between 18 and 80 years, of both sexes, regardless of race and/or ethnicity, a diagnosis of PD according to Movement Disorders criteria, up to grade 2 on the Hoehn & Yarh scale^20^ , with regular medical follow-up, and a Montreal Cognitive Assessment (MoCA-B)^21^ score of 22 or higher at the time of recruitment. The inclusion criterion for both PD groups was a MoCA-B score of 22 or higher at the time of recruitment. However, in the DBS group, minor deviations from this threshold were observed at the time of data collection, likely due to postoperative cognitive fluctuations. A small number of participants (eight) with scores slightly below 22 were retained after a qualitative clinical review, which considered preserved functional independence, as evidenced by scores on the Activities of Daily Living (ADL) scale; the absence of self-reported cognitive complaints; and performance consistent with the group average on other cognitive measures, ensuring a representative sample without compromising methodological consistency. The exclusion criteria were color blindness, personality disorders and cognitive disorders diagnosis. 

 The inclusion criteria for the pairing healthy group were as follows: literate people, aged between 18 and 80 years, of both sexes, regardless of race and/or ethnicity, without a diagnosis of PD or other neurodegenerative and psychiatric diseases, who received regular medical follow-up, agreed with the informed consent form, and a MoCA-B^21^ score of 22 or higher. The exclusion criteria for the healthy control group were people with personality disorders, suspected PD, cognitive disorders, color blindness, and those who had suffered a stroke at some point in their lives. For all three groups, data related to personality, psychiatric, and medical conditions were collected through self-report measures by the medical team and no formal diagnoses were made at the time of recruitment (Table 1). 

**Table 1:** Demographic data of the sample.

	Healthy control (n=23) M (SD)	Drug (n=23) M (SD)	DBS (n=23) M (SD)	p-value
Sex, count, males, n (%)	12 (52%)	12 (52%)	16 (70%)	
Age, years	67.6 (6.6)	66.1 (11.2)	64.5 (6.6)	0.304
Education, years	14 (3.7)	12 (4.7)	12.3 (4.4)	0.212
Disease time, years		8.7 (4.4)	17 (6.8)	<0.001*
Surgery time, years			6.5 (4.3)	
LEDD, mg		1,101 (804)	1,280 (585)	0.414
ADL, score	27 (0)	24.8 (2.4)	22.3 (3.3)	<0.001*
BDI-II, score	9.3 (7.1)	14.7 (7.6)	20.2 (11)	<0.001*
QUIP-RS, score	28.4 (12.1)	40.9 (18.5)	44.1 (14.2)	<0.001*
SAS, score	8.8 (5)	13.2 (5.1)	16.5 (7.8)	<0.001*
MoCA, score	26.6 (1.9)	23.1 (4.5)	22 (5)	<0.001*

Abbreviations: LEDD, Levodopa Equivalent Daily Dose; Drug, Parkinson’s disease patients treated only with drugs; DBS, Parkinson’s disease patients treated with Deep Brain Stimulation; M, Mean; SD, Standard Deviation; ADL, Activities Daily Living; BDI, Beck Depression Inventory; QUIP-RS, Questionnaire for Impulsive-Compulsive Disorders in Parkinson’s Disease; SAS, Starkstein Apathy Scale; MoCA: Montreal Cognitive Assessment.

Note: *Statistically significant.

### Instruments

#### The identification protocol

 This protocol allows obtaining general patient data such as age, sex, education, clinical history, disease duration and time since surgery. 

#### Activities of Daily Living (ADL)^22^

 This assessment is composed of ten questions with three possible answers: without help, with partial help and unable. A higher score indicates greater independence in daily activities, whereas lower scores indicate increased dependence on others for assistance. 

#### The Beck Depression Inventory-II (BDI-II)^23^

 This instrument was used to score and categorize the severity of depressive symptoms. Generally, a score of 0–13 indicates no/or minimal depression symptoms, 14–19 indicates mild depression, 20–28 indicates moderate depression and 29–63 indicates severe depression. 

#### The Questionnaire for Impulsive-Compulsive Disorders in Parkinson’s Disease (QUIP-RS)^24^

 It is a self-administered scale with 28 questions assessing gambling, shopping, eating, sexual behavior, punding, and hobbyism. Each item is rated on a 5-point Likert scale, with scores ranging from 0 (never) to 4 (very often), resulting in a total score ranging from 0 to 112. 

#### The Starkstein Apathy Scale (SAS)^25^

 This scale quantifies the symptoms of apathy through 14 items that include emotional, cognitive, and behavioral dimensions. It is composed of a Likert scale of four alternatives, ranging from 0 (not at all) to 4 (extremely), with some items being scored inversely, and the total score ranging from 0 to 42. 

#### The Montreal Cognitive Assessment (MoCA-B)^21^

 This assessment evaluates five cognitive functions (memory, language, capacity for abstraction, visuospatial capacity, and concentration). It takes 10–15 minutes to complete, and the maximum score is 30 points, with scores below 22 indicating possible cognitive dysfunction^26^ . 

#### The Trail Making Test Parts A and B (TMT-A and TMT-B)^27^

 This is a neuropsychological assessment tool used to evaluate cognitive flexibility and attentional control. The goal is to rapidly and accurately complete the task. Performance is measured by completion time. 

#### The Verbal Fluency Test^28^

 This is a cognitive assessment tool used to evaluate language and frontal lobe functions. It requires the participant to generate words from a given category (animals and fruits) or letters (F, A, and S) within a set time limit (60 seconds). The final score is determined by the number of words generated per category within one minute. 

#### The Stroop Test^29^

 This is a widely used cognitive assessment that measures selective attention and inhibitory control. Participants complete three tasks: reading color words, reading neutral words (random words not associated with colors, such as "never," "today," "each," and "everything"), and naming the ink color while ignoring the written word. The test score was determined by the time taken to complete the third task (the interference task). 

#### WAIS-III Digits Subtest^30^

 This test is used to assess attention, immediate and working memory. In the first part, the individual is asked to repeat the sequence of numbers given by the examiner in the same order. The second part consists of repeating the numbers in reverse order. The final score is based on the number of successful attempts. 

#### The NEO-FFI-R Personality Inventory^31^

 This is used to assess personality. It is a 60 items self-reported questionnaire, with a 5-point scale ranging from "strongly disagree" to "strongly agree", based on the FFM. The scoring involves summing the items for each factor, converting the raw scores into T-scores, and generating a personality profile, which is performed by the test scoring software. 

### Procedures

 This study was approved by the Ethics Committee of the Federal University of Paraná (No. 5.609.208), and data collection was conducted at a specialized PD association in Curitiba, Brazil. It started with explaining the research objectives and collecting the necessary signatures for participation agreements (informed consent form). After the signature, the identification protocol, ADL, and MoCA were applied. Based on the results of this instrument (MoCA), we proceeded with the testing by applying, in sequence: BDI-II, QUIP-RS, SAS, WAIS-III digits subtest direct and reverse order, semantic and phonological fluency tests, TMT-A, TMT-B, Stroop Test, and finally, the NEO-FFI-R. Only one participant could not complete the MoCA test and did not follow through with the assessment. The healthy control group had no relationship with the PD patients evaluated. 

### Data analysis

 All statistical analyses were conducted using Jamovi Statistics Software^32^ , and the alpha was set at p<0.05. Shapiro-Wilk were performed to assess the distribution of the data. Independent chi-square test, ANOVA, and Tukey’s post-hoc test were used to analyze all variables. Z-score was calculated to classify the performance on the instruments. A multivariate stepwise linear analysis was conducted to assess the association between demographic data, neuropsychiatric variables, cognitive performance, and personality factors (Appendix 1 and 2, available at  https://www.demneuropsy.org/wp-content/uploads/2025/06/DN-2024.0254-Supplementary-Material.pdf). This approach also allowed us to control for potential confounding variables such as age, education, disease duration, and neuropsychiatric measures when analyzing group differences. 

## RESULTS

Table 1 shows the comparative results between the control and PD groups (Drug and DBS) for the studied variables. Sociodemographic variables included sex, age, education, disease and surgery duration, and Levodopa Equivalent Daily Dose. The ADL, BDI-II, QUIP-RS, SAS and MoCA results tests are also presented. Data are presented as mean, standard deviation for continuous variables and frequency (%) for categorical variables, as well as p-values. 

Table 2 shows the performance of cognitive assessments. In the Animal Verbal Fluency Test [F (2,43)=14.2; p<0.001] and the TMT-B [F (2,39)=12.8; p<0.001], a difference was found between the healthy control group and the other two groups. In the FAS Verbal Fluency Test [F (2,42)=5.59; p=0.007] and Stroop test [F (2,36)=8.17; p=0.001], a difference was found between the healthy control group and the DBS group. 

**Table 2:** Executive functions assessment.

	Healthy control (n=23) M (SD)	Drug (n=23) M (SD)	DBS (n=23) M (SD)	p-value
Digits inverse	4.7 (1.7)	4.1 (1.8)	3.9 (1.7)	0.295
VF – Animals	18.9 (3.7)	14.8 (4.8)	12.3 (4.7)	<0.001*
VF – FAS	36.1 (12.7)	28.7 (15.8)	24.5 (10.6)	0.007*
Stroop 3 time	36.7 (11.3)	53.6 (25.2)	60 (33.1)	0.001*
TMT-B	100.2 (59.8)	188.9 (113.4)	219.2 (111)	<0.001*

Abbreviations: Drug, Parkinson’s Disease patients treated only with drugs; DBS, Parkinson’s Disease patients treated with Deep Brain Stimulation; M, Mean; SD, Standard Deviation; VF, Verbal Fluency; Stroop 3 time, Total time taken to complete the third stage of the Stroop test TMT, Trial Making Test.

Note: *Statistically significant.

Table 3 shows the results of the personality assessment. Among the five factors, only two showed no significant differences between the groups: neuroticism [F (2,43)=0.98; p=0.38] and openness [F (2,42)=1.71; p=0.19]. For agreeableness [F (2,43)=5.08; p=0.01] and conscientiousness [F (2,43)=7.68; p=0.001], the healthy control group diverged from the other two groups. In extraversion [F (2,43)=7.99; p=0.001], the DBS group showed a significant difference compared to the control and drug groups. 

**Table 3:** Personality assessment.

	Healthy control (n=23) M (SD)	Drug (n=23) M (SD)	DBS (n=23) M (SD)	p-value
Neuroticism	23.6 (6.1)	24.7 (5.6)	26.3 (6.7)	0.381
Extraversion	29.8 (5.2)	27.6 (5.4)	23 (6.2)	0.001*
Openness	29.8 (4.8)	27.6 (7.3)	27.1 (5.2)	0.192
Agreeableness	35.5 (5.5)	31.6 (4.7)	31 (4.3)	0.010*
Conscientiousness	36.3 (6.1)	31.5 (6)	29.7 (5.3)	0.001*

Abbreviations: Drug, Parkinson’s Disease patients treated only with drugs; DBS, Parkinson’s Disease patients treated with Deep Brain Stimulation; M, Mean; SD, Standard Deviation.

Notes: *Statistically significant.

Table 4 presents the three models constructed using multivariate stepwise linear analysis. In Model 1, sociodemographic variables explained 49% of the ADL variability (R^2^=0.49), with significant associations to disease duration, surgery duration, and education. For BDI-II (R ^2^=0.34), significant associations were found with disease duration and education. For QUIPRS, sociodemographic variables explained 32% of the variance, with associations to disease duration and education. For SAS, the explanation was 21%, with a significant association to surgery time. For MoCA, 19% of the variance was explained, with an association to disease duration. 

**Table 4:** Standard Coefficient (beta) in multiple regression analyses of four models.

	Model 1					Model 2					Model 3					Model 4				
	ADL	BDI-II	QUIP	SAS	MOCA	ADL	BDI-II	QUIP	SAS	MOCA	ADL	BDI-II	QUIP	SAS	MOCA	ADL	BDI-II	QUIP	SAS	MOCA
Sociodemographic data																				
Disease time	-0.44*	0.55*	0.40*	0.16	-0.34*	-0.29*	0.44*	0.43*	-0.22	0.06	-0.28*	0.48*	0.51*	-0.24	0.13	-0.27*	0.39*	0.48*	-0.14	0.14
Surgery time	-0.24*	-0.08	-0.007	0.29*	-0.02	-0.14	-0.18	-0.03	0.16	0.04	-0.09	-0.21	-0.08	0.19	0.03	-0.12	-0.22	-0.01	0.03	0.02
Age	-0.007	-0.10	-0.14	-0.003	0.03	-0.01	-0.11	-0.15	0.02	-0.02	-0.03	-0.09	-0.09	<0.001	0.06	-0.08	0.03	-0.11	-0.06	0.05
Education	0.25*	-0.21*	-0.32*	-0.18	0.21	0.14	-0.14	-0.31*	0.02	-0.01	0.07	-0.16	-0.35*	0.04	-0.04	0.11	-0.11	-0.36*	0.03	-0.07
Neuropsychiatric data																				
ADL							-0.12	0.06	-0.57*	0.35*		-0.09	0.06	-0.59*	0.01		-0.06	0.06	-0.57*	0.01
BDI-II						-0.07		-0.18	0.20	-0.26*	-0.05		-0.18	0.21	-0.05	-0.04		-0.14	0.08	-0.04
QUIP-RS						0.03	-0.15		0.05	-0.24*	0.03	-0.15		0.07	-0.13	0.03	-0.10		-0.02	-0.17
SAS						-0.35*	0.20	0.06		-0.004	-0.33*	0.20	0.08		-0.02	-0.42*	0.08	-0.03		-0.09
MoCA						0.18*	-0.22*	-0.24*	-0.004		0.02	-0.11	-0.32	-0.05		0.01	-0.06	-0.38*	-0.14	
Cognitive data																				
Inverse digits											0.11	-0.01	-0.03	0.001	-0.10	0.10	-0.03	-0.06	-0.05	-0.12
VF animals											0.18	0.04	-0.04	0.11	0.14	0.15	0.02	-0.01	0.17	0.17
FAS total											-0.02	-0.01	0.14	-0.09	0.15	0.04	-0.07	0.23	0.13	0.23
Stroop 3 time											-0.04	0.21	0.06	-0.06	-0.37*	-0.09	0.14	0.003	-0.11	-0.35*
TMT-B											-0.03	-0.02	-0.17	0.01	-0.40*	-0.02	-0.006	-0.13	0.01	-0.38*
Personality data																				
Neuroticism																0.05	0.34*	-0.13	-0.04	-0.08
Extraversion																0.05	-0.12	0.24	0.003	<0.001
Openness																-0.27*	0.09	-0.05	-0.39*	-0.04
Agreeableness																-0.07*	0.08	0.15	-0.02	0.07
Conscientiousness																0.03	-0.17	-0.42*	-0.31*	-0.16
R^2^	0.49	0.34	0.32	0.21	0.19	0.66	0.47	0.37	0.45	0.37	0.70	0.49	0.39	0.46	0.75	0.74	0.65	0.50	0.65	0.77

 In Model 2, the inclusion of neuropsychiatric variables increased the model’s explanatory value to 66%, with significant associations between ADL, disease duration, SAS, and MoCA. Additional associations were found with BDI-II, disease duration, and MoCA. For QUIP-RS, R^2^ increased to 0.37, with significant associations to disease duration, education, and MoCA. For SAS, R^2^ rose to 0.45, with a significant association to ADL. For MoCA, R^2^ of 0.37 showed further associations with ADL, BDI-II, and QUIP-RS. 

 In Model 3, adding cognitive variables increased R^2^ to 0.70, with significant associations between ADL, disease duration, and SAS. For BDI-II, an association with disease duration was found (R^2^=0.49). For QUIP-RS, R ^2^ increased to 39%, with associations to disease duration and education. For SAS, R^2^ rose to 46%, with a significant association only to ADL. For MoCA, explanatory power increased to 75%, with associations to the Stroop test and TMT-B. 

 In the final model, adding personality variables increased R^2^ to 0.74 for ADL, with significant associations to disease duration, SAS, and openness. For BDI-II, R^2^ of 0.65 showed associations with disease duration and neuroticism. For QUIP-RS (R^2^=0.50), significant associations were found with disease duration, education, MoCA, and conscientiousness. For SAS, R^2^ rose to 0.65, with significant associations to ADL, openness, and conscientiousness. With MoCA, R^2^ increased to 0.77, with associations to the Stroop test and TMT-B. 

## DISCUSSION

 This study aimed to investigate the effects of STN-DBS on personality and EF in PD patients. To the best of our knowledge, this is the first study to concurrently examine these domains. Our findings indicated no significant differences in EF or personality between the DBS and drug-only groups, as both groups exhibited comparable cognitive and personality profiles. Nonetheless, both treatment groups showed significant differences from healthy controls, a finding consistent with prior research suggesting that PD progression affects the cognitive and personality domains. These findings imply that neuropsychiatric and cognitive factors may play a more substantial role in influencing PD-related changes than STN-DBS intervention alone, highlighting the need for further investigation into the impact of various treatments on these outcomes. 

 These results must account for the natural progression of PD, which often leads to cognitive decline and dementia in patients at the moderate or advanced stages^33^ . Previous studies have found significant associations between depressive symptoms, age, disease duration, and ADL, supporting the present findings^34^ . Additionally, some medications can induce impulsivity, gambling, and hypersexuality, which may also impact the study results, as it remains unclear whether these effects are due to disease progression or medication^35^ . 

 Our results are consistent with those of a previous study by Wu et al.^33^ , Dong et al.^36^ , and Witt et al.^37^ , which reported no significant cognitive impairment in patients who underwent DBS, aligning with our cross-sectional findings. Dong et al. reported no alterations in the brain areas related to executive control in PD patients^36^ . Wu et al. reviewed the effects of STN-DBS on cognitive function and observed that executive functions remained stable in the intermediate postoperative stage (1–2 years), though they tended to decline in other stages^33^ . Witt et al. found no significant differences in global cognitive function, verbal memory, working memory, and attention in patients who underwent DBS when compared with those receiving medical treatment^37^ . 

 By contrast, Georgiev et al.^8^ , Xie et al.^18^ and Maheshwary et al.^38^ , reported longitudinal evidence of cognitive decline in patients undergoing DBS, which differs from the cross-sectional profile observed in our study^8,18,38^. Georgiev et al. reported that apathy and cognitive function deteriorated beyond preoperative levels in DBS patients^8^ . Maheshwary et al. conducted a systematic review of 13 studies and found minor yet significant cognitive side effects, including executive dysfunction and decreased attention^38^ . Xie et al. performed a meta-analysis of ten studies (three randomized and seven non-randomized controlled trials) and found that chronic STN stimulation could lead to subtle declines in global cognition, memory, phonemic fluency, semantic fluency, and executive function^18^ . It is important to emphasize that, due to its cross-sectional design, our study cannot track cognitive changes over time or establish causal relationships. Therefore, comparisons with longitudinal studies or meta-analyses should be interpreted with caution. 

 Regarding personality, our study’s findings are consistent with some research showing low levels of agreeableness, conscientiousness, and extraversion in PD patients^39-42^. PD patients are often more cautious and introverted^43^ , and these traits are sometimes associated with depression^ 39,44^. In our study, the PD groups scored lowest in these traits, with a weak association with depression. Conscientiousness is typically a protective factor linked to the organization and informed behavior that avoids risky health practices^45^ . Low agreeableness was associated with competitiveness, antagonism, and egocentricity^42^ , and negatively correlated with neuroticism and depression. 

 Several studies have examined differences between PD patients treated with medication alone versus those treated with DBS, or assessed changes before and after surgery^5, 46,47^. Some authors suggest that personality changes following DBS are more likely to involve characteristic adaptations rather than dispositional traits, although role-specific behaviors in daily life and work, social, and leisure activities can also be affected^5^ . Thomson et al. conducted a prospective qualitative study evaluating patients before and after surgery and noted changes in personality post-DBS^47^ . Lhommée et al. conducted neurological and neuropsychological assessments one month before and 12 months after surgery, highlighting the impact of STN-DBS and dopaminergic treatment on personality traits in PD patients^46^

 The discrepancies between our cross-sectional findings and previous longitudinal studies or meta-analyses on cognitive impairment following DBS may be explained by several methodological and sample-related differences. First, meta-analyses often include studies with diverse methodologies, follow-up durations, and diagnostic criteria, which may lead to varying conclusions. In contrast, our study utilized a specific sample of patients with PD and a standardized methodology, potentially explaining some of these differences. Additionally, variability among DBS patients, such as differences in age, disease stage, and individual characteristics, could contribute to divergent findings. The natural progression of PD itself can influence cognitive function independently of DBS, and, although we attempted to account for this, its impact may not be fully captured. Finally, the effects of DBS on cognitive function can be influenced by the duration of follow-up and the settings of DBS parameters, which vary across studies. These methodological and sample-related factors may account for the observed discrepancies, and future research with more homogeneous samples and extended follow-up periods could provide further clarity. In spite of this, our study contributes valuable new insights, particularly through the detailed analysis of personality and cognitive changes associated with DBS using the FFM. This structured approach offers a more nuanced evaluation of personality traits than has been previously explored. Additionally, it highlights associations that have not been previously assessed in this manner, contributing significantly to the body of literature. This advancement serves as an important guide for patients with PD, helping them better understand the potential changes in their personal and cognitive characteristics. 

 Despite certain methodological limitations, this study provides valuable insights into the effects of DBS on personality traits and cognitive performance in individuals with PD. Among these limitations are the relatively small sample size and the inclusion of eight participants with MoCA scores slightly below the original cutoff — a justified decision based on clinical criteria, although it may introduce some degree of heterogeneity. Although the sample size was determined using power analysis, the limited number of participants per group may reduce the ability to detect subtle differences, particularly in personality domains. Another important limitation is the comparison of our cross-sectional findings with results from longitudinal studies and meta-analyses. Since cross-sectional designs provide only a snapshot in time, unlike longitudinal studies that allow tracking changes over time, such comparisons should be interpreted with caution and do not permit causal inferences. Nevertheless, by concurrently examining personality and executive function using a structured, theory-driven framework (FFM), this study offers novel contributions to the literature and lays the groundwork for future prospective research with pre- and post-operative assessments, which are necessary to better understand the long-term impact of DBS on personality and cognition. 

 In conclusion, our study highlights the complexity of PD-related cognitive and personality changes, reinforcing the importance of neuropsychiatric factors in understanding disease progression. While DBS does not appear to significantly alter these domains compared to medication alone, further longitudinal research is required to confirm these observations. To the best of our knowledge, this is the first study to concurrently examine these domains. These findings emphasize the need to consider the natural progression of PD, which is often characterized by cognitive decline and personality changes, offering valuable insights into the complex interaction of these factors in shaping the clinical profile of PD patients. 

## Data Availability

The datasets generated and/or analyzed during the current study are publicly available at:  https://osf.io/m5gxw/?view_only=710fce5af2d74e1cb2306fc91b67451d

## References

[1] Lin Z, Zhang C, Li D, Sun B (2021). Lateralized effects of deep brain stimulation in Parkinson’s disease: evidence and controversies. NPJ Parkinsons Dis.

[2] Armstrong MJ, Okun MS (2020). Diagnosis and treatment of Parkinson disease: a review. JAMA.

[3] Castonguay AM, Gravel C, Lévesque M (2021). Treating Parkinson’s disease with antibodies: previous studies and future directions. J Parkinsons Dis.

[4] Zesiewicz TA (2019). Parkinson disease. Continuum.

[5] Wilt JA, Merner AR, Zeigler J, Montpetite M, Kubu CS (2021). Does personality change follow deep brain stimulation in Parkinson’s disease patients?. Front Psychol.

[6] Fox SH, Katzenschlager R, Lim SY, Barton B, Bie RMA, Seppi K (2018). International Parkinson and movement disorder society evidence-based medicine review: update on treatments for the motor symptoms of Parkinson’s disease. Mov Disord.

[7] Arten TLS, Hamdan AC (2022). Executive functions and memory in Parkinson’s disease patients with Deep Brain Stimulation. Aging Health Res.

[8] Georgiev D, Mencinger M, Rajnar R, Mušič P, Benedičič M, Flisar D (2021). Long-term effect of bilateral STN-DBS on non-motor symptoms in Parkinson’s disease: a four-year observational, prospective study. Parkinsonism Relat Disord.

[9] Brezovar S, Pažek L, Kavčič M, Georgiev D, Trošt M, Flisar D (2022). Personality changes after subthalamic nucleus stimulation in Parkinson’s sisease. J Parkinsons Dis.

[10] Pham U, Solbakk AK, Skogseid IM, Toft M, Pripp AH, Konglund AE (2015). Personality changes after deep brain stimulation in Parkinson’s disease. Parkinsons Dis.

[11] Costa PT, McCrae RR (1992). Four ways five factors are basic. Pers Individ Dif.

[12] Mezquita L, Bravo AJ, Morizot J, Pilatti A, Pearson MR, Ibáñez MI (2019). Cross-cultural examination of the Big Five Personality Trait Short Questionnaire: measurement invariance testing and associations with mental health. PLoS One.

[13] Morizot J (2014). Construct validity of adolescents’ self-reported big five personality traits: importance of conceptual breadth and initial validation of a short measure. Assessment.

[14] Arten TLS, Hamdan AC (2023). Five-factor model of personality and Parkinson’s Disease: a systematic review. Geriatr Gerontol Aging.

[15] Santangelo G, Garramone F, Baiano C, D’Iorio A, Piscopo F, Raimo S (2018). Personality and Parkinson’s disease: a meta-analysis. Parkinson Relat Disord.

[16] Rossi M, Bruno V, Arena J, Cammarota A, Merello M (2018). Challenges in PD patient management after DBS: a pragmatic review. Mov Disord Clin Pract.

[17] Højlund A, Petersen MV, Sridharan KS, Østergaard K (2017). Worsening of verbal fluency after deep brain stimulation in Parkinson’s Disease: a focused review. Comput Struct Biotechnol J.

[18] Xie Y, Meng X, Xiao J, Zhang J, Zhang J (2016). Cognitive changes following bilateral deep brain stimulation of subthalamic nucleus in Parkinson’s Disease: a meta-analysis. Biomed Res Int.

[19] Faul F, Erdfelder E, Buchner A, Lang AG (2009). Statistical power analyses using G*Power 3.1: Tests for correlation and regression analyses. Behav Res Methods.

[20] Hoehn MM, Yahr MD (1967). Parkinsonism: onset, progression, and mortality. Neurology.

[21] Nasreddine ZS, Phillips NA, Bédirian V, Charbonneau S, Whitehead V, Collin I (2005). The Montreal Cognitive Assessment, MoCA: a brief screening tool for mild cognitive impairment. J Am Geriatr Soc.

[22] Lawton MP, Brody EM (1969). Assessment of older people: self-maintaining and instrumental activities of daily living. Gerontologist.

[23] Beck AT, Ward CH, Mendelson M, Mock J, Erbaugh J (1961). An inventory for measuring depression. Arch Gen Psychiatry.

[24] Guerra DF, Silva AEL, Paz TSR, Filho LNS, Vasconcellos LF, Britto VLS (2020). Measurement properties from the Brazilian Portuguese version of the QUIP-RS. NPJ Parkinsons Dis.

[25] Starkstein SE, Mayberg HS, Preziosi TJ, Andrezejewski P, Leiguarda R, Robinson RG (1992). Reliability, validity, and clinical correlates of apathy in Parkinson’s disease. J Neuropsychiatry Clin Neurosci.

[26] Amatneeks TM, Hamdan AC (2019). Montreal Cognitive Assessment for cognitive assessment in chronic kidney disease: a systematic review. J Bras Nefrol.

[27] Bowie CR, Harvey PD (2006). Administration and interpretation of the Trail Making Test. Nat Protoc.

[28] Benton AL, Hamsher KS (1976). Multilingual aphasia examination: manual of instructions.

[29] Stroop JR (1935). Studies of interference in serial verbal reactions. J Exp Psychol.

[30] Wechsler D (1997). Wechsler adult intelligence scale.

[31] Costa PT, McCrae RR (1989). NEO PI/FFI manual supplement for use with the NEO personality inventory and the NEO five-factor inventory.

[32] The Jamovi Project (2022). Jamovi (version 2.3) [Internet].

[33] Wu B, Han L, Sun BM, Hu XW, Wang XP (2014). Influence of deep brain stimulation of the subthalamic nucleus on cognitive function in patients with Parkinson’s disease. Neurosci Bull.

[34] Arten TLS, Hamdan AC (2024). Depressive symptoms in Brazilian Parkinson’s disease patients treated with subthalamic nucleus deep brain stimulation : a cross-sectional study. Appl Neuropsychol Adult.

[35] Voon V, Napier TC, Frank MJ, Sgambato-Faure V, Grace AA, Rodriguez-Oroz M (2017). Impulse control disorders and levodopa-induced dyskinesias in Parkinson’s disease: an update. Lancet Neurol.

[36] Dong W, Qiu C, Jiang X, Shen B, Zhang L, Liu W (2020). Can the executive control network be used to diagnose Parkinson’s Disease and as an efficacy indicator of deep brain stimulation?. Parkinsons Dis.

[37] Witt K, Daniels C, Reiff J, Krack P, Volkmann J, Pinsker MO (2008). Neuropsychological and psychiatric changes after deep brain stimulation for Parkinson’s disease: a randomised, multicentre study. Lancet Neurol.

[38] Maheshwary A, Mohite D, Omole JA, Bhatti KS, Khan S (2020). Is deep brain stimulation associated with detrimental effects on cognitive functions in patients of Parkinson’s disease? A systematic review. Cureus.

[39] Damholdt MF, Østergaard K, Borghammer P, Larsen L (2011). The parkinsonian personality and concomitant depression. J Neuropsychiatry Clin Neurosci.

[40] Glosser G, Clark C, Freundlich B, Kliner-Krenzel L, Flaherty P, Stern M (1995). A controlled investigation of current and premorbid personality: characteristics of Parkinson’s disease patients. Mov Disord.

[41] McRae C, Cherin E, Diem G, Vo AH, Ellgring JH, Russell D (2003). Does personality change as a result of fetal tissue transplantation in the brain?. J Neurol.

[42] Sachdeva J, Harbishettar V, Barraclough M, McDonald K, Leroi I (2014). Clinical profile of compulsive sexual behaviour and paraphilia in Parkinson’s disease. J Parkinsons Dis.

[43] Aasly A, Aasly JO (2021). Writers are common among Parkinson’s disease patients: a longitudinal study. Behav Neurol.

[44] Damholdt MF, Callesen MB, Møller A (2014). Personality characteristics of depressed and non-depressed patients with Parkinson’s disease. J Neuropsychiatry Clin Neurosci.

[45] Terracciano A, Löckenhoff CE, Zonderman AB, Ferrucci L, Costa PT (2008). Personality predictors of longevity: activity, emotional stability, and conscientiousness. Psychosom Med.

[46] Lhommée E, Boyer F, Wack M, Pélissier P, Klinger H, Schmitt E (2017). Personality, dopamine, and Parkinson’s disease: Insights from subthalamic stimulation. Mov Disord.

[47] Thomson CJ, Segrave RA, Racine E, Warren N, Thyagarajan D, Carter A (2020). "He’s back so i’m not alone": the impact of deep brain stimulation on personality, self, and relationships in Parkinson’s disease. Qual Health Res.

